# Thymosin α1 combined with XELOX improves immune function and reduces serum tumor markers in colorectal cancer patients after radical surgery

**DOI:** 10.1515/biol-2022-0793

**Published:** 2024-04-13

**Authors:** Li Sha, Hao Zhang, Xiwei Zhang

**Affiliations:** Department of General Surgery, Shuguang Hospital affiliated to Shanghai University of Traditional Chinese Medicine, Shanghai 200025, China

**Keywords:** thymosin α1, oxaliplatin, capecitabine, colorectal cancer, immune function, tumor marker, radical surgery

## Abstract

This study aimed to investigate the efficacy of thymosin α1 combined with XELOX in improving immune function and reducing serum tumor markers in patients with colorectal cancer (CRC) after radical surgery. A total of 180 patients who underwent radical surgery for CRC were divided into two groups: an observation group (*n* = 94) receiving thymosin α1 in combination with XELOX and a control group (*n* = 86) receiving XELOX alone. Immune function, inflammatory factor levels, serum tumor markers, and quality of life were assessed before and after treatment. Adverse reactions and recurrence rates were compared between the two groups in 1 and 3 years. Following therapy, there was a notable increase in the levels of CD3+, CD4+, and CD4+/CD8+ in all cohorts, particularly in the observation cohort, when compared to pre-therapy levels. Conversely, CD8+ levels decreased across all cohorts, especially in the observation cohort. Additionally, there was an increase in the levels of IL-2 and IFN-γ in the observation cohort, compared to both pre-therapy and control cohort levels, while IL-6 levels decreased. The presence of CEA, CA242, and CA724 reduced significantly across all cohorts following post-therapy, particularly in the observation cohort. Post-therapy, there was a significant increase in the scoring for role, cognitive, social, emotional, and somatic functions in all cohorts, with the most significant improvement observed in the observation cohort. There were no significant differences in the incidence of side effects across cohorts, while neutropenia events were significantly lower in the observation cohort (32.98%) compared to the control cohort (48.84%). The 12-month recurrence rate showed no statistical significance across cohorts, while the observation cohort had a significantly lower three-year recurrence rate (24.47%) compared to the control cohort (59.30%). Thymosin α1 combined with XELOX is effective in improving immune function, reducing serum tumor markers, and minimizing recurrence in CRC patients after radical surgery. This combination therapy may be a promising new direction for the treatment of CRC.

## 1 Introduction

Colorectal cancer (CRC) is a common and highly lethal malignant tumor in the digestive system, which typically affects middle-aged and elderly individuals. Early-stage CRC often presents with nonspecific symptoms such as diarrhea and constipation. As the disease progresses, patients may experience clinical symptoms such as rectal bleeding, changes in bowel habits, and changes in stool consistency. Although the specific etiology of CRC remains unclear, it is generally believed to result from the synergistic effect of environmental factors, dietary habits, genetic factors, and other factors. In recent years, changes in people’s dietary habits and lifestyles have led to a gradual increase in the incidence of CRC [1]. Currently, newly diagnosed cases of CRC worldwide account for 10.0% of all cancer cases, making CRC the third most common malignant tumor globally and the second leading cause of cancer-related deaths. Therefore, it is urgent to adopt effective treatment measures to reduce the mortality of CRC, prolong patient survival time, and improve the quality of life (QOL) in clinical practice.

Radical surgery is currently an effective way to treat CRC. However, nearly 50% of cases experience postoperative recurrence and metastasis, which is an important reason for the treatment failure of metastatic and recurrent CRC. Therefore, postoperative adjuvant chemotherapy is important for reducing the risk of tumor recurrence and metastasis. XELOX, consisting of capecitabine and oxaliplatin, is a first-line treatment for CRC. Capecitabine is a fluoropyrimidine derivative that can be quickly absorbed by the intestinal mucosa and converted into 5-FU through thymidine phosphorylase. This increases drug concentration within the tumor, accelerates tumor cell apoptosis, and achieves an anticancer effect [2]. Oxaliplatin is a third-generation platinum-based drug that can form intra- and interstrand cross-linking complexes with tumor DNA through covalent bonding, interfere with DNA synthesis, and affect cell division, thus inhibiting the proliferation of tumor cells. It can also increase the activity of thymidine phosphorylase and enhance the pharmacological effect of capecitabine [3]. The synergy of both drugs can better exert the anticancer effect. However, existing studies have shown that chemotherapy drugs have a killing effect on both tumor cells and immune cells but may also cause severe immune dysfunction in patients, which is highly likely to induce tumor cell proliferation. XELOX may also have different degrees of impact on the patient’s immunity, thereby affecting the patient’s chemotherapy tolerance and prognosis [4,5]. Therefore, how to balance the tumor, drug, and body’s immunity is currently a hot topic in cancer treatment.

Thymosin alpha-1 is a bioactive polypeptide composed of 28 amino acids, which is isolated and purified from thymosin fraction 5 of the thymus gland. It is generated by enzymatic degradation of thymosin pro-alpha in the body. This small molecule exhibits high biological activity and immunostimulatory effects, and it regulates and enhances immune function within the body. As an immune modulator, thymosin alpha-1 can induce the transformation of T cells in the thymus into mature T lymphocytes, which further differentiate into functionally diverse T cell subpopulations and constantly expand them. Moreover, research has demonstrated that thymosin alpha-1 also has a regulatory effect on inflammation and immune tolerance. The immunomodulatory effect of thymosin alpha-1 strengthens the body’s cell-mediated immunity and enables it to directly or indirectly act on tumor cells, ultimately leading to the killing of tumor cells [6]. Currently, thymosin alpha-1 is applied in immunotherapy for severe hepatitis and has been found to play a crucial role in immunotherapy for lung tumors, liver cancer, and other tumors [7,8]. In addition, the combination of thymosin alpha-1 with cisplatin and fluorouracil-based neoadjuvant chemotherapy can elevate T lymphocyte and NK cell levels in patients with esophageal cancer, relieve systemic inflammation, and reduce grade 3–4 adverse events [9]. Therefore, the application of thymosin alpha-1 in the immunotherapy of CRC represents a promising new direction for treatment.

The combination of XELOX with other drugs, such as HLX04 and bevacizumab, has garnered considerable attention among researchers for the treatment of CRC. In an effort to enhance medical management and further improve the control efficacy of CRC, this study administered thymosin α1 in combination with XELOX to cases undergoing radical surgery for CRC. The aim was to investigate the effects of this combination on cases’ immune function and postoperative serum tumor markers

## 2 Data and methods

### 2.1 Research subjects

The inclusion criteria for this study were established as follows: (1) meeting the diagnostic criteria for CRC [10], as confirmed by imaging and pathology; (2) a single lesion without metastasis; and (3) complete clinical data.

The exclusion criteria were: (1) cases with intestinal obstruction or intestinal perforation; (2) cases with acute cardiac or renal diseases; (3) cases with severe postoperative infection; (4) cases with a history of malignant tumors; (5) cases with immune dysfunction or severe blood diseases; and (6) cases with allergic constitution.

**Figure 1:**
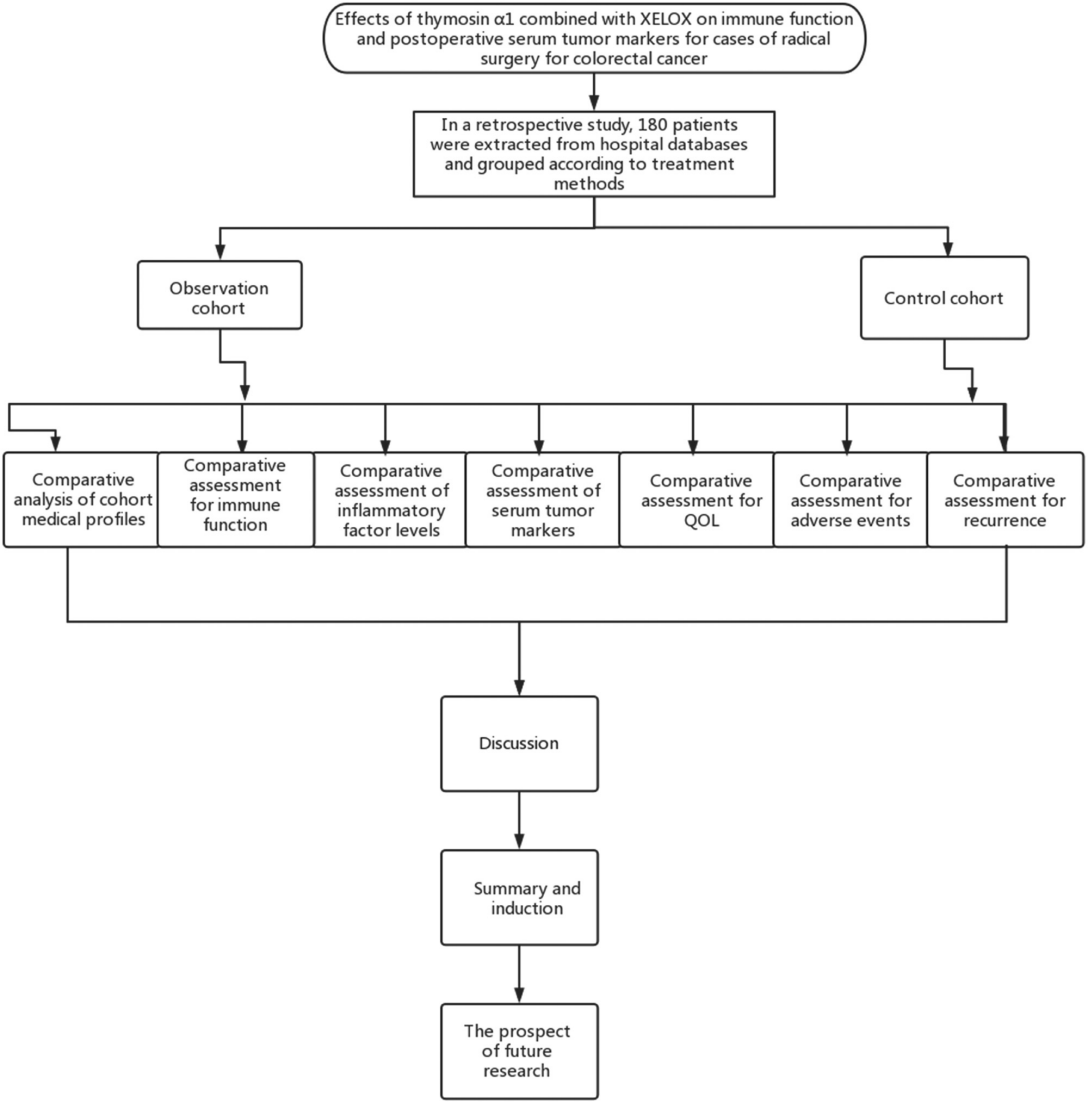
The flowchart showing the research process and methodology applied in the present study.

This study was a retrospective cohort study conducted at a single center. Using the aforementioned criteria, a total of 180 patients who underwent radical surgery for CRC at our institute between January 2017 and December 2019 were selected and separated into two cohorts: an observation cohort (*n* = 94) and a control cohort (*n* = 86), based on different post-surgical therapeutic strategies. There were no significant differences found in the clinical profiles of the observation and control cohorts (*P* > 0.05). The research process of the article has been illustrated in a flowchart, as shown in Figure 1. A comparative analysis of cohort medical profiles is presented in Table 1.

**Table 1:** Comparative analysis of medical profiles among the cohorts

Cohort	Number of cases	Gender (male/female, cases)	Age (years)	BMI (kg/m^2^)	Location of lesions		TNM stage		Histological type		
					Colon cancer	Rectal cancer	Ⅱ	Ⅲ	Adenocarcinoma	Mucinous adenocarcinoma	Undifferentiated carcinoma
Observation cohort	94	51/43	55.39 ± 6.99	22.88 ± 3.89	25	69	67	27	62	22	10
Control cohort	86	44/42	54.49 ± 7.67	21.90 ± 4.39	23	63	55	31	51	28	7
*χ* ^2^/*t*		0.172	0.828	1.583	0.001		1.103		1.969		
*P*		0.678	0.409	0.115	0.982		0.294		0.374		


**Informed consent:** Informed consent has been obtained from all individuals included in this study.
**Ethical approval:** The research related to human use has been complied with all the relevant national regulations, institutional policies and in accordance with the tenets of the Helsinki Declaration, and has been approved by the authors’ institutional review board or equivalent committee.

### 2.2 Treatment method

Both the observation and control cohorts received their first chemotherapy treatment half a month after surgery. The control cohort was treated with XELOX, which consists of intravenous oxaliplatin (Jiangsu Aosaikang Pharmaceutical Co., LTD., National drug approval number: H20064296) at a dose of 130 mg/m^2^ on the 1st day, once a day, and intravenous capecitabine (Qilu Pharmaceutical Co., LTD., National Drug Approval number: H20133361) at a dose of 1,000 mg/m^2^ on the 1st to 14th day, twice a day. The second course of chemotherapy was performed 7 days after drug withdrawal, and a total of four cycles of chemotherapy were administered to both cohorts.

The observation cohort received thymosin α1 (Xian Disai Biological Pharmaceutical, National drug approval number: H20003484) in combination with XELOX. Thymosin α1 was subcutaneously injected three times per week at a dose of 1.6 mg, while XELOX was administered according to the same protocol as in the control cohort. Both cohorts received a total of four cycles of chemotherapy.

### 2.3 Data collection and comparison

The study employed the following comparative analyses across both cohorts:

(1) Comparative of immune function: A total of 3 mL of venous blood was collected from subjects before and after therapy. The supernatant was obtained after anticoagulation with EDTA and centrifugation. The percentages of CD3+, CD4+, and CD8+ cells were evaluated using a flow cytometer from BD Company of the United States, with CD4+/CD8+ ratio calculated.

(2) Comparative analysis of inflammatory factor expression: Venous blood (3 mL) was collected before and after therapy, with the serum separated after centrifugation. Serum interleukin (IL)-2, IL-6, and interferon-γ (IFN-γ) were analyzed using enzyme-linked immunosorbent assay (ELISA).

(3) Comparative analysis of serum tumor biomarkers: Serum levels of carcinoembryonic antigen (CEA), carbohydrate antigen 242 (CA242), and carbohydrate antigen 724 (CA724) were evaluated using ELISA before and after therapy.

(4) Comparative analysis of QOL [11]: The EORTC QLQ-C30 QOL questionnaire assessed five dimensions: cognitive, physical, emotional, role, and social functions. Increased scores indicated improved QOL. The questionnaire demonstrated a Cronbach’s α coefficient of 0.81.

(5) Comparative analysis of adverse events.

(6) Comparative assessment of reoccurrence rate: The recurrence and metastasis at 1 and 3 years after operation were compared between the two groups. The recurrence and metastasis of patients were obtained through telephone follow-up at 3 months, 6 months, 1 year, 2 years, and 3 years after discharge.

### 2.4 Statistical methods

SPSS 20.0 statistical software was used for statistical analysis. The measurement data were expressed as *x̅* ± *s* To validate the normality and homogeneity of variance in the sample data, we employed the Kolmogorov–Smirnov test in combination with exploratory descriptive statistics. For normally distributed data, we used the *t*-test, whereas non-normally distributed data were tested using the *U*-test. The counting data were expressed as rate (%), and *χ* ^2^ was used for comparison. *P* < 0.05 was considered to be statistically significant.

## 3 Results

### 3.1 Comparative evaluation of immune function

**Figure 2:**
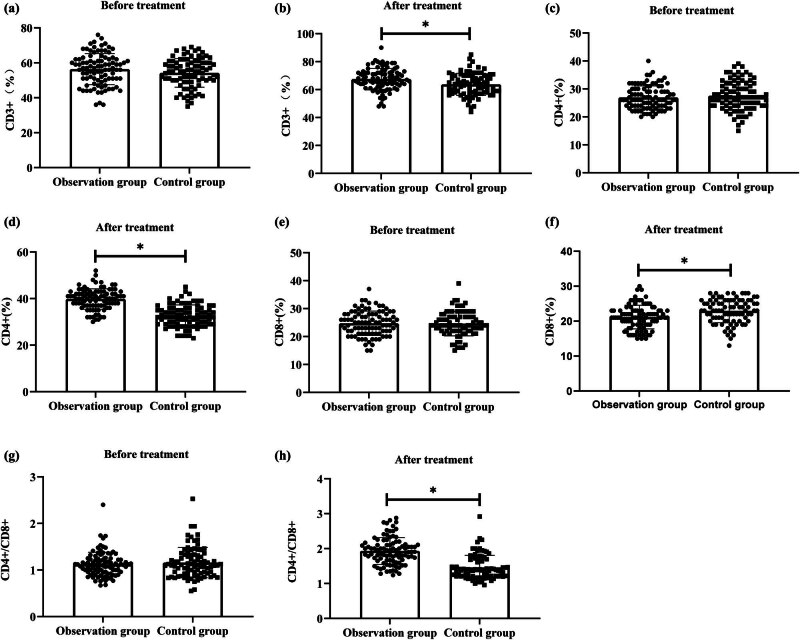
Comparative evaluation of immune function. (a) CD3+ before treatment; (b) CD3+ after treatment; (c) CD4+ before treatment; (d) CD4+ after treatment; (e) CD8+ before treatment; (f) CD8+ after treatment; (g) CD4+/CD8+ before treatment; and (h) CD4+/CD8+ after treatment.

Both the observation and control groups demonstrated increases in CD3+, CD4+, and CD4+/CD8+ after treatment compared to pre-treatment levels. In the observation group, CD3+ increased from 56.39 ± 8.87 to 67.50 ± 7.74, CD4+ increased from 26.96 ± 4.10 to 39.81 ± 4.38, and CD4+/CD8+ increased from 1.12 ± 0.26 to 1.93 ± 0.38. In the control group, CD3+ increased from 54.23 ± 8.21 to 63.71 ± 7.92, CD4+ increased from 27.57 ± 5.18 to 32.61 ± 4.66, and CD4+/CD8+ increased from 1.16 ± 0.33 to 1.46 ± 0.35. Collectively, the observation group had significantly higher levels of CD3+, CD4+, and CD4+/CD8+ compared to the control group (*P* < 0.05) (Figure 2).

Both the observation and control groups showed a decrease in CD8+ levels after treatment. In the observation group, CD8+ levels decreased from 24.75 ± 4.35 to 21.16 ± 3.38, while in the control group, the level decreased from 24.60 ± 4.38 to 22.95 ± 3.42. Notably, the observation group had significantly lower levels of CD8+ compared to the control group (*P* < 0.05) (Figure 2).

### 3.2 Comparative evaluation of inflammatory factor levels

**Figure 3:**
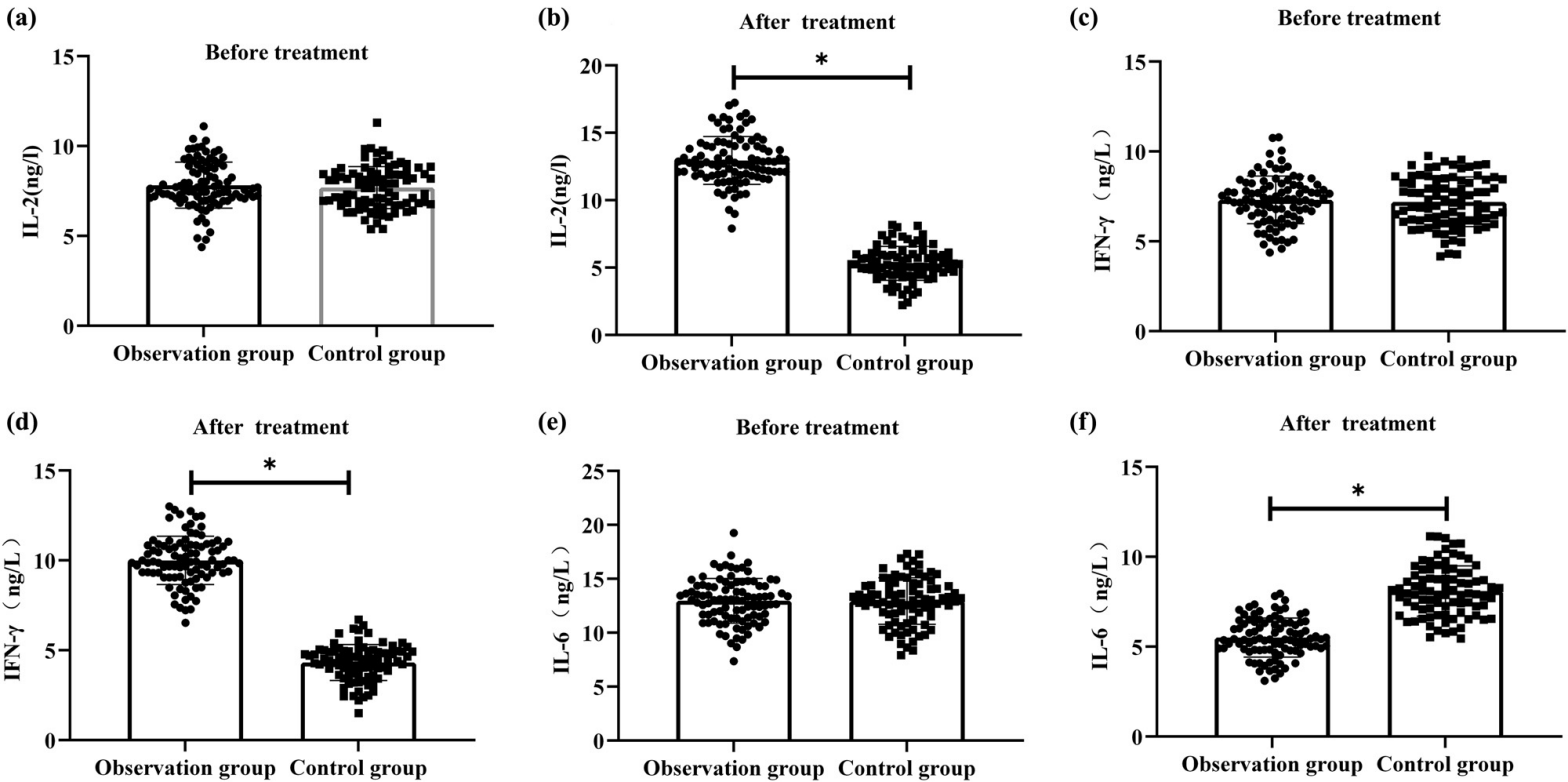
Comparative evaluation of levels of inflammatory factors. (a) IL-2 before treatment; (b) IL-2 after treatment; (c) IFN-γ before treatment; (d) IFN-γ after treatment; (e) IL-6 before treatment; and (f) IL-6 after treatment.

In the observation group, IL-2 and IFN-γ levels significantly increased after treatment compared to pre-treatment levels (*P* < 0.05). Specifically, the IL-2 level increased from 7.82 ± 1.29 to 12.95 ± 1.78, and the IFN-γ level increased from 7.69 ± 1.17 to 5.31 ± 1.26. However, in the control group, both IL-2 and IFN-γ levels significantly decreased after treatment compared to pre-treatment levels (*P* < 0.05). The IL-2 level decreased from 7.69 ± 1.17 to 5.31 ± 1.26, and the IFN-γ level decreased from 7.69 ± 1.17 to 4.32 ± 1.00. The observation group had significantly higher levels of IL-2 and IFN-γ compared to the control group (*P* < 0.05) (Figure 3a and b).

After treatment, both the observation and control groups exhibited decreased levels of IL-6 compared to pre-treatment levels. In the observation group, IL-6 levels decreased from 12.96 ± 2.08 to 5.51 ± 1.08, while in the control group, the levels decreased from 12.94 ± 2.16 to 8.10 ± 1.40. The observation group had significantly lower levels of IL-6 compared to the control group (*P* < 0.05). (Figure 3c).

### 3.3 Comparative evaluation of serum tumor markers

**Figure 4:**
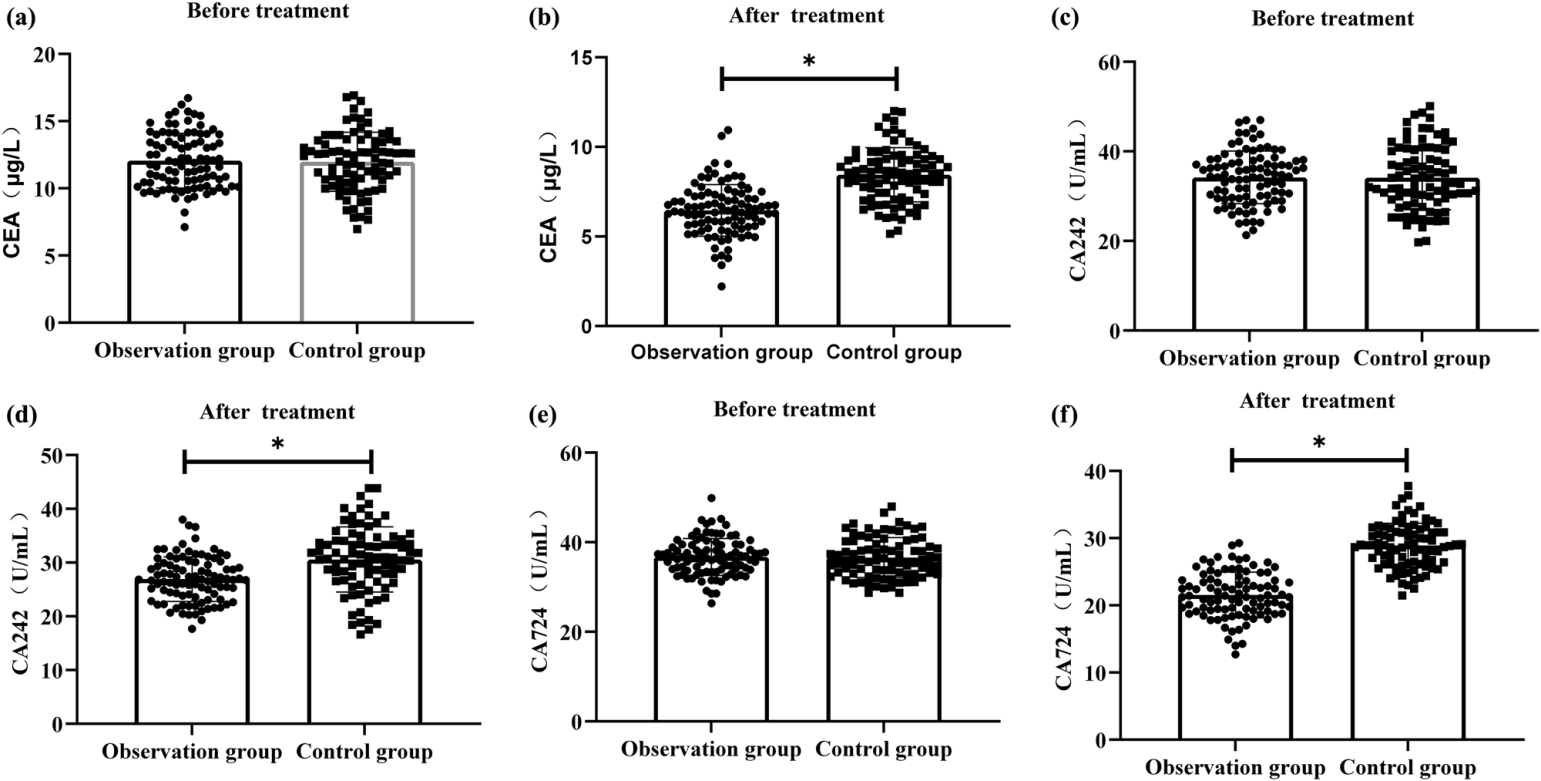
Comparative evaluation of serum tumor markers. (a) CEA before treatment; (b) CEA after treatment; (c) CA242 before treatment; (d) CA242 after treatment; (e) CA724 before treatment; and (f) CA724 after treatment.

After treatment, both the observation and control groups exhibited decreased levels of serum CEA, CA242, and CA724 compared to pre-treatment levels. In the observation group. The CEA level decreased from 12.06 ± 2.03 to 6.46 ± 1.44, the CA242 level decreased from 34.19 ± 5.86 to 26.92 ± 4.16, and the CA724 level decreased from 36.71 ± 4.11 to 21.55 ± 3.39. The control group also exhibited decreased levels of CEA, CA242, and CA724 after treatment. Specifically, the CEA level decreased from 11.98 ± 2.20 to 8.44 ± 1.51, the CA242 level decreased from 34.12 ± 7.13 to 30.58 ± 6.07, and the CA724 level decreased from 36.58 ± 4.46 to 28.90 ± 3.33. These differences were all statistically significant. However, the observation group had significantly higher levels of changes in CEA, CA242, and CA724 compared to the control group (*P* < 0.001) (Figure 4).

### 3.4 Comparative evaluation of QOL

After treatment, the levels of scorings for role, cognitive, social, emotional, and physical functions across cohorts in both the observation and control groups increased compared to pre-treatment levels. In the observation group, the role function level increased from 50.96 ± 7.01 to 68.83 ± 6.05, the cognitive function level increased from 66.63 ± 6.97 to 78.43 ± 6.50, the social function level increased from 51.27 ± 5.21 to 59.48 ± 5.74, the emotional function level increased from 55.83 ± 6.09 to 64.53 ± 6.82, and the physical function level increased from 60.27 ± 6.49 to 71.90 ± 6.99.

Similarly, in the control group, the role function level increased from 51.53 ± 7.46 to 68.83 ± 6.05, the cognitive function level increased from 64.91 ± 6.67 to 72.27 ± 6.61, the social function level increased from 50.69 ± 6.55 to 55.19 ± 5.24, the emotional function level increased from 54.83 ± 5.56 to 60.38 ± 5.94, and the physical function level increased from 61.03 ± 7.12 to 66.72 ± 7.40.

**Figure 5:**
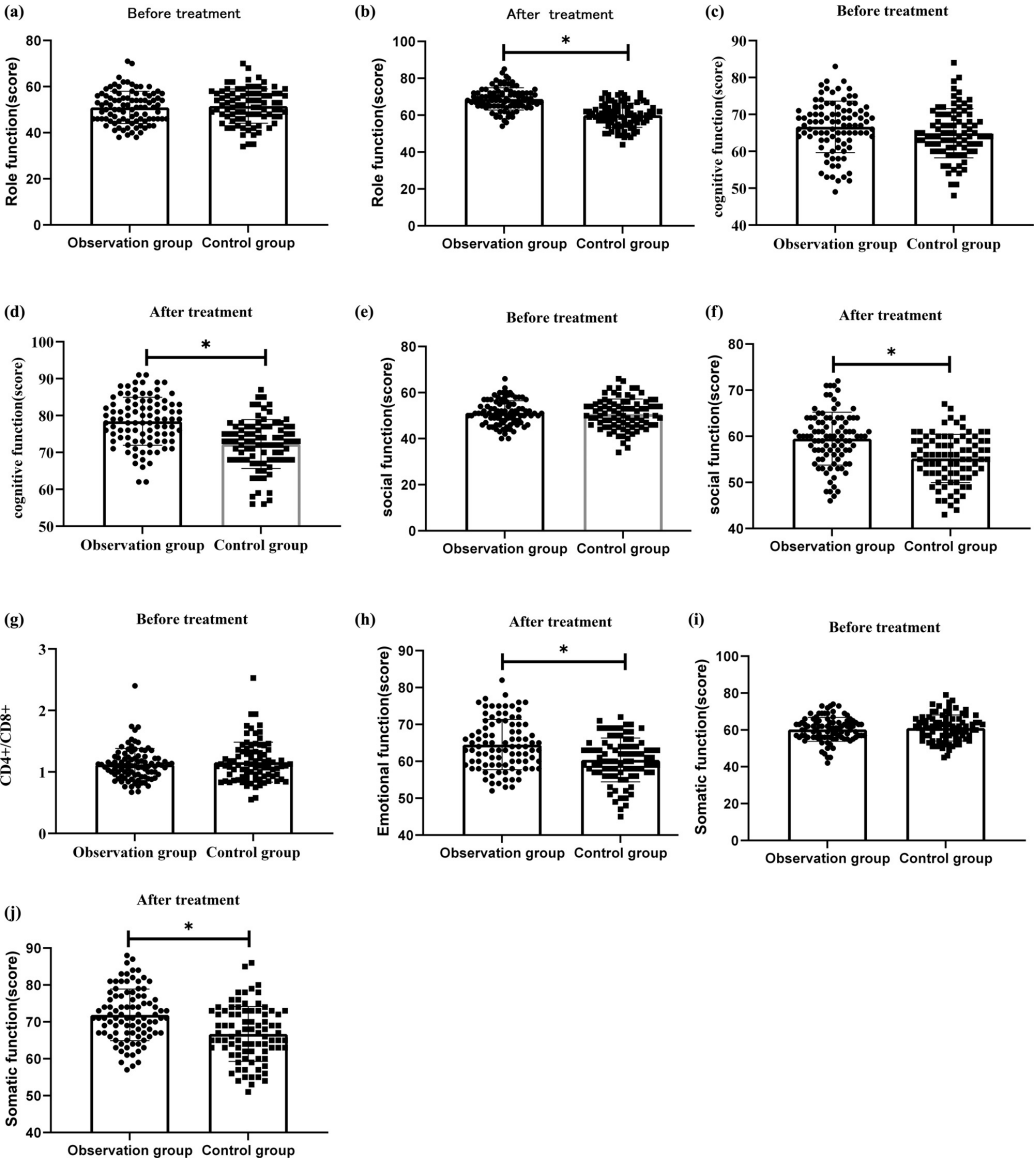
Comparative evaluation of QOL. (a) role function before treatment; (b) role function after treatment; (c) cognitive function before treatment; (d) cognitive function after treatment; (e) social function before treatment; (f) social function after treatment; (g) CD4+/CD8+ before treatment; (h) emotional function after treatment; (i) somatic function before treatment; and (j) somatic function after treatment.

These differences were all statistically significant. Furthermore, the observation group had significantly higher changes in scorings for role, cognitive, social, emotional, and physical functions compared to the control group (*P* < 0.001) (Figure 5).

### 3.5 Comparative evaluation of adverse events

Regarding the incidence of bone marrow suppression, nausea and vomiting, abnormal hepatic function, thrombocytopenia, and diarrhea, no statistical significance was observed across cohorts (*P* > 0.05). However, the incidence of neutropenia within the observation cohort was 32.98%, while it was 48.84% within the control cohort, which was statistically significant (*P* < 0.05) (Table 2).

**Table 2:** Comparative evaluation of adverse events (*n*, %) among the cohorts

Cohort	Number of cases	Bone marrow suppression	Nausea and vomiting	Abnormal liver function	Thrombocytopenia	Neutropenia	Diarrhea
Observation cohort	94	1 (1.06)	50 (53.19)	14 (14.89)	27 (28.72)	31 (32.98)	18 (19.14)
Control cohort	86	2 (2.32)	49 (56.98)	13 (15.12)	23 (26.74)	42 (48.84)	21 (24.42)
*χ* ^2^		0.436	0.260	0.002	0.088	4.685	0.735
*P*		0.509	0.610	0.967	0.767	0.030	0.391

### 3.6 Comparative evaluation of recurrence

The 1-year recurrence rates of the observation and control cohorts were 14.89 and 25.58%, respectively, but without being statistically significant (*P* > 0.05). However, the 3-year recurrence rate within the observation cohort was 24.47, while it was 59.30% within the control cohort, which was statistically significant (*P* < 0.05) (Table 3).

**Table 3:** Comparative evaluation of recurrence (*n*, %) among the cohorts

Cohort	Number of cases	1-Year recurrence rate	3-Year recurrence rate
Observation cohort	94	14 (14.89)	23 (24.47)
Control cohort	86	22 (25.58)	51 (59.30)
*χ* ^2^		3.206	22.510
*P*		0.073	＜0.001

## 4 Discussion

Thymosin α1, initially used for immunotherapy of severe hepatitis, has recently been found to be beneficial in immunotherapy of lung tumors, liver cancer, and other types of tumors [12]. Several studies [13] indicate that when combined with cisplatin and fluorouracil neoadjuvant chemotherapy, thymosin α1 can increase the levels of T lymphocytes and NK cells in esophageal cancer cases, alleviate inflammation, and decrease grade 3 and 4 adverse events. After radical resection of CRC, patients’ immune function is impaired, and chemotherapy alone is often insufficient to achieve the expected results. Therefore, it is crucial to enhance patients’ immune ability. Thymosin α1 can improve patients’ immune function, and when combined with chemotherapy, it is a common clinical treatment for cancer. XELOX is a common chemotherapy treatment for CRC. Therefore, in this study, thymosin α1 combined with XELOX was used as adjuvant therapy after CRC surgery.

The development and progression of tumors in the body are intricately linked to cellular immune function. T lymphocytes express the CD3+ surface marker, which consists of six peptide chains. These cells can be classified into two subgroups, namely cognitive function and CD8+. CD3+ T cells play a critical role in eradicating tumors, and a balanced ratio of cognitive function to CD8+ is essential for maintaining normal immune function. When tumor-mass growth affects and inhibits lymphocyte DNA synthesis, an imbalance of cognitive function to CD8+ occurs, which impairs the body’s immunity [14,15]. Cognitive function is crucial for killing tumor cells by secreting Th1 and Th2 molecules. Th1 secretes IL-2 and IFN-γ among other factors, which positively regulate immune function. Conversely, Th2 secretes IL-6 and other factors that may negatively affect immunity by influencing the transcriptional activity of tumor cells through the AKT signaling pathway [16].

In the context of this study, post-therapy observations of the cohort receiving thymosin α1 combined with XELOX showed increased levels of CD3+, cognitive function, cognitive function/CD8+, IL-2, and IFN-γ compared to the pre-therapy levels. On the other hand, CD8+ and IL-6 levels decreased compared to the control cohort. These findings suggest that thymosin α1 combined with XELOX can improve postoperative immune function in patients with CRC by regulating the secretion of inflammatory factors. Thymosin α1 is a polypeptide substance that inhibits steroid-induced apoptosis of thymocytes, regulates the differentiation and maturation of T cells, activates CD3+ cells, strengthens mixed lymphocyte reactions, and increases the number of cognitive function cells, thereby improving cellular immune function. Therefore, thymosin α1 plays a crucial role in the anti-tumor effect of XELOX [17,18]. Nevo et al. [19] have demonstrated that thymosin α1 can enhance the effectiveness of intraperitoneal hyperthermic perfusion chemotherapy and induce an anti-tumor immune response of Th1, thereby improving the survival rate of CRC mice with peritoneal metastasis.

Tumor markers play a crucial role in assessing tumor progression within the body. CEA, a glycoprotein with a molecular weight of 200 kDa, is commonly found in embryonic mucosal cells. Under normal conditions, its concentration is low in the body. However, during malignant tumor development, its level significantly increases. Therefore, CEA levels are useful in diagnosing and prognosing breast, ovarian, esophageal cancers, and other related diseases [20,21]. CA242 is a mucin antigen present in normal pancreatic and colonic mucosa. However, its levels increase significantly in malignant tumors of the digestive tract [22]. CA724 is an orosomucoid molecule that has high diagnostic value for gastrointestinal tumors and ovarian cancer [23].

Post-therapy observations during this investigation showed that the levels of CEA, CA242, and CA724 were lower in the observation cohort compared to pre-therapy levels. Additionally, the observation cohort that received thymosin α1 combined with XELOX showed reduced levels of CEA, CA242, and CA724 compared to the control cohort. These findings suggest that thymosin α1 can potentially reduce serum tumor marker levels in patients with CRC cases in combination with XELOX. Thymosin α1 can reduce the immunosuppression caused by chemotherapy and accelerate the recovery of T lymphocytes, thus helping XELOX to inhibit the growth and reproduction of tumor blood vessels, which improves the *in vivo* microenvironment and downregulates serum tumor biomarkers [24,25].

A subsequent investigation has revealed that the observation cohort had higher post-therapy scores for all QOL dimensions compared to the control cohort. Although the 1-year recurrence rate did not differ significantly between the cohorts, the 3-year recurrence rate in the observation cohort was notably lower (24.47%) than the rate in the control cohort (59.30%). These findings confirm that the combination of Thymosin α1 with XELOX can improve the QOL of CRC patients and decrease disease recurrence. This may be attributed to the treatment’s ability to more effectively enhance immunity and reduce inflammation, promote postoperative recovery, and lower the recurrence rate. The improvement in role, cognition, social, emotional, and physical functions in the QOL indicates that the appropriate medication regimen can effectively enhance the physiological function and QOL of patients who have undergone radical resection of CRC. The improvement in the physiological state can significantly impact the patient’s QOL.

Thymosin α1 can be used as an adjuvant to radiotherapy and chemotherapy in tumor treatment, with no significant adverse effects found in multiple clinical trials[26]. This study found no significant difference in the incidence of myelosuppression, nausea and vomiting, abnormal liver function, thrombocytopenia, and diarrhea between the cohorts. However, the incidence of neutropenia in the observation cohort (32.98%) was lower than the rate in the control cohort (48.84%). These findings suggest that thymosin α1 can enhance the body’s immunity and reduce the occurrence of neutropenia without increasing other adverse events.

However, this study has some limitations, including its retrospective nature, small sample size, and potential selection bias. Therefore, a prospective study incorporating multiple centers and expanding the sample size is necessary in the future to mitigate these limitations. In the future, the combination of different drugs based on immunotherapy combined with chemotherapy can also be attempted to explore the best treatment plan for patients after radical resection of CRC.

In summary, the utilization of a combination therapy consisting of thymosin α1 and XELOX has demonstrated significant benefits for patients with CRC who are undergoing radical surgery. This treatment approach has been shown to improve postoperative immune function, reduce levels of inflammatory factors and serum tumor markers, and enhance the QOL of patients. Additionally, this protocol has been associated with a reduced recurrence rate and a favorable safety profile. The contribution and novelty of this study lie in the development of a new treatment protocol for the management of CRC following radical surgery.
